# Image generator for tabular data based on non-Euclidean metrics for CNN-based classification

**DOI:** 10.1371/journal.pone.0340005

**Published:** 2026-01-09

**Authors:** Yu-Rong Lin, Han-Ming Wu

**Affiliations:** Department of Statistics, National Chengchi University, Taipei City, Taiwan, Republic of China; Naval Postgraduate School, UNITED STATES OF AMERICA

## Abstract

Tabular data is the predominant format for statistical analysis and machine learning across domains such as finance, biomedicine, and environmental sciences. However, conventional methods often face challenges when dealing with high dimensionality and complex nonlinear relationships. In contrast, deep learning models, particularly Convolutional Neural Networks (CNNs), are well-suited for automatic feature extraction and achieve high predictive accuracy, but are primarily designed for image-based inputs. This study presents a comparative evaluation of non-Euclidean distance metrics within the Image Generator for Tabular Data (IGTD) framework, which transforms tabular data into image representations for CNN-based classification. While the original IGTD relies on Euclidean distance, we extend the framework to adopt alternative metrics, including one minus correlation, Geodesic distance, Jensen-Shannon distance, Wasserstein distance, and Tropical distance. These metrics are designed to better capture complex, nonlinear relationships among features. Through systematic experiments on both simulated and real-world genomics datasets, we compare the performance of each distance metric in terms of classification accuracy and structural fidelity of the generated images. The results demonstrate that non-Euclidean metrics can significantly improve the effectiveness of CNN-based classification on tabular data. By enabling a more accurate encoding of feature relationships, this approach broadens the applicability of CNNs and offers a flexible, interpretable solution for high-dimensional, structured data across disciplines.

## Introduction

In recent years, deep learning has achieved remarkable advancements, with Convolutional Neural Networks (CNNs) [1,2] demonstrating exceptional predictive performance across a wide range of tasks, including object detection, image classification, and natural language processing. CNNs are specifically designed to process grid-like data structures, such as images, leveraging efficient feature extraction, the identification of nonlinear correlations and higher-order patterns, and a compact architecture enabled by weight sharing. Despite their success in image-based tasks, CNNs face significant challenges when applied to tabular data, which consists of rows representing observations and columns corresponding to variables (features). Unlike images, tabular data lacks an inherent spatial structure, making it difficult for CNNs to leverage their spatial feature extraction capabilities effectively. Moreover, CNNs often suffer from interpretability issues, limiting their application in fields where explainability is crucial, such as healthcare, finance, and scientific research.

Traditional statistical methods and machine learning techniques, such as Linear Discriminant Analysis (LDA), Principal Component Analysis (PCA), and Support Vector Machines (SVM), have long served as foundational approaches for analyzing and predicting tabular data. These methods typically involve fitting statistical models based on assumptions about the underlying structure and relationships between variables. While effective in certain scenarios, they often struggle to capture complex nonlinear interactions and high-dimensional feature dependencies, leading to suboptimal feature extraction and reduced predictive accuracy. In contrast, deep learning models, particularly CNNs, have demonstrated superior performance in learning intricate feature representations but are inherently designed for spatial data rather than structured tabular datasets.

To bridge this gap and leverage the power of CNNs for tabular data analysis, recent research has explored tabular-to-image conversion techniques, which transform high-dimensional tabular data into image-like representations (referred to as pseudo-images in this study). This transformation enables CNNs to process tabular datasets more effectively by encoding meaningful relationships between features into spatial representations. As a result, these techniques have demonstrated improved predictive accuracy and enhanced analytical capabilities (e.g., [3]). A brief review of these methods is provided in the next section.

The growing significance of tabular-to-image conversion techniques highlights their potential to integrate deep learning with structured data across various domains. If effectively adapted, CNNs could offer substantial benefits for tabular data classification tasks. Building upon this foundation, this study seeks to enhance the Image Generator for Tabular Data (IGTD) [4] by addressing its key limitations and extending its applicability to CNN-based classification. IGTD relies on Euclidean distance to organize tabular features within a 2D grid. However, this approach does not adequately consider the high-dimensional characteristics, nonlinear structures, and complex feature distributions present in tabular data. To address these limitations, we introduce four alternative non-Euclidean distance metrics, one minus correlation, Geodesic distance, Jensen-Shannon distance, Wasserstein distance, and Tropical distance, to more effectively capture the underlying structure of high-dimensional data. By adopting these metrics, our study aims to enhance both the interpretability and predictive performance of CNN-based classification models for tabular data.

This article proceeds as follows: A review of related work on tabular-to-image conversion methods is first presented. We then describe the IGTD algorithm, followed by our proposed improvements using non-Euclidean distance metrics. The empirical evaluation, covering both simulation studies and real-world datasets, is presented next. Finally, we conclude by summarizing the key findings and discussing their broader implications.

## Advancements in tabular-to-image conversion for CNN-based predictions

The transformation of tabular data into image representations suitable for deep learning has gained increasing attention in recent years. This approach enables Convolutional Neural Networks (CNNs) and other image-based deep learning architectures to be directly applied to structured tabular datasets, eliminating the need for modifications to CNN architectures. One of the key advantages of tabular-to-image conversion is its ability to preserve spatial relationships between variables, allowing CNNs to leverage their hierarchical feature extraction capabilities. When tabular data is encoded as pseudo-images, CNNs can effectively extract complex patterns and inter-variable relationships, potentially improving classification accuracy. Furthermore, this transformation facilitates the application of additional deep learning algorithms designed for image data, such as deep neural networks (DNNs), transfer learning models, and autoencoders. However, redesigning CNN architectures specifically for tabular data, as explored by [5–8], is beyond the scope of this study. Instead, this work focuses on leveraging tabular-to-image conversion techniques to enhance CNN-based classification while preserving the structural integrity of the original data.

The early exploration of tabular-to-image conversion for CNNs was pioneered by Lyu and Haque (2018) [9], who mapped high-dimensional RNA-Seq data into two-dimensional images by aligning genes according to their chromosomal positions. Their approach demonstrated high classification accuracy when processed by CNNs. Similarly, Ma and Zhang (2018) [10] introduced OmicsMapNet, which employed the Treemap algorithm [11] to hierarchically structure genomic data into 2D images. However, this method required prior biological knowledge to spatially organize molecular features into meaningful patterns. A major milestone in this domain was the introduction of DeepInsight [12], a generalizable framework for converting non-image data, such as RNA-Seq, text, and artificial datasets, into images. Unlike its predecessors, DeepInsight leveraged CNNs without requiring domain-specific knowledge for feature arrangement. Building on this progress, Bazgir et al. (2020) [13] proposed REFINED-CNN (Representation of Features as Images with Neighborhood Dependencies), which employed Bayesian Metric Multidimensional Scaling (BMDS) to transform tabular features into compact image representations while preserving their spatial relationships.

Subsequent advancements focused on enhancing interpretability. PathCNN [14] integrated multi-omics data and pathway information using PCA, while SurvCNN [15] applied nonlinear dimensionality reduction techniques such as t-SNE and UMAP to reduce image dimensions. Zhu et al. (2021) [4] introduced IGTD (Image Generator for Tabular Data), a technique designed to preserve the neighborhood structure of data features by arranging them using Euclidean distances. IGTD optimizes variable placement by minimizing Euclidean distances before projecting them onto a two-dimensional grid, improving classification accuracy when applied to CNNs. This study highlighted the significant impact of distance metrics on feature arrangement and classification outcomes, motivating further exploration of alternative distance metrics to enhance predictive performance within the IGTD framework. Further refinements in tabular-to-image conversion include Vec2Image [16], which transforms high-dimensional biological omics data into 2D images while incorporating feature abundance and correlation. Wang et al. (2022) [17] proposed a multi-index sorting method for gene expression data, enabling image-based survival prediction in lung cancer patients and extending the applicability of these techniques beyond classification tasks. Tab2Vox [18] introduced a novel approach that converts tabular data into 3D voxel images, allowing 3D CNNs to capture hierarchical feature representations.

Recent contributions have continued to refine these methodologies. TINTO [19] transforms structured tabular data into images using PCA and t-SNE while incorporating fuzzification techniques to enhance feature extraction and generalization. TablEye [20] addresses the challenge of few-shot learning in tabular data by integrating tested few-shot learning algorithms with embedding functions, enabling effective learning with limited labeled data. MWCapsNet [21] employs multi-level wavelet decomposition and capsule networks to extract multi-scale spatial and frequency features, improving classification performance in imbalanced datasets. Binary Image Encoding (BIE) [22] transforms binary network traffic data into images using one-hot encoding, mitigating the challenges posed by the lack of inherent order in categorical features. Matsuda et al. (2024) [23] classified tabular-to-image conversion methods into three categories: prior knowledge-based, feature permutation-based, and dimensionality reduction-based approaches. They also identified key limitations, including a lack of consideration for prediction loss during image generation, potential non-interpretability, and the inclusion of irrelevant features. To address these challenges, they proposed HACNet, a hard attention-based tabular-to-image converter that selectively filters important variables before transformation, significantly improving interpretability and predictive performance compared with existing methods such as DeepInsight, REFINED-CNN, and IGTD.

Other studies have extended tabular-to-image conversion techniques to time series and survival analysis applications (e.g., [15,24–29]). These techniques typically convert time series data into recurrence plots, Gramian angular fields, or Markov transition fields, effectively encoding both local and global temporal relationships in a visual format to leverage CNNs for pattern recognition and forecasting.

Recent efforts have also focused on optimizing spatial representations and feature encoding to further enhance CNN-based classification. Notable examples include MRep-DeepInsight [30], which dynamically maps feature vectors using multiple manifold techniques to improve model robustness; TabMap [31], which encodes feature values as pixel intensities to create spatially semantic topographic maps; and Tensorized Image Generator (TIG) [32], which emphasizes feature relationships using intersecting diagonal lines on a grid. OmicsFootPrint [33] presents an innovative approach to transforming omics data into circular images organized by genomic locations, offering customizable and visually interpretable representations.

Additionally, several methods have focused on feature encoding strategies to enhance classification outcomes. Tab2Visual [34] represents features using vertical bars with proportional widths and colors, incorporating novel augmentation techniques such as elastic distortion and morphological operations. Table2Image [35] employs an autoencoder architecture to generate realistic and interpretable images while preserving the original characteristics of tabular data. Similarly, AutoIRAD [36] integrates dimensionality reduction and dataset-specific image representation to facilitate robust classification across datasets with varying dimensions. Other studies have explored advanced preprocessing techniques and distance metrics to further optimize classification performance. For instance, LM-IGTD [37] enhances low-dimensional tabular data representation by introducing stochastic noise and dynamically adjusting feature dimensions. Fuzzy Convolutional Neural Network (FCNN) [38] applies fuzzification techniques to map feature values into fuzzy memberships on an image canvas, improving CNN-based classification in uncertain or imprecise data environments. Finally, we note that Jiang et al. (2025) [39] have recently conducted a comprehensive survey and empirical benchmarking of representation learning for tabular data, offering valuable insights into the relative strengths of existing tabular-to-image conversion strategies.

Despite the significant advancements in tabular-to-image conversion techniques for CNN-based classification, several challenges remain. Most existing methods rely on Euclidean distance-based feature arrangements, which may not effectively capture the complex, nonlinear structures present in high-dimensional tabular datasets. Exploring alternative non-Euclidean distance metrics, such as one minus correlation, Geodesic, Jensen-Shannon, Wasserstein distances, or Tropical distance, may provide more accurate and meaningful spatial representations, ultimately improving both model interpretability and classification performance.

## The image generator for tabular data (IGTD)

Zhu et al. (2021) [4] introduced IGTD (Image Generator for Tabular Data), a novel approach for converting tabular data into image representations, enabling deep learning with Convolutional Neural Networks (CNNs) without requiring domain-specific knowledge. IGTD constructs a two-dimensional grid, where each cell corresponds to a specific data point in the table. The grid is then populated with color intensities proportional to the values of the corresponding data points. To optimize the grid layout, IGTD assigns features to pixels in a manner that minimizes the discrepancy between the ranking of distances among features in the original tabular space and the ranking of distances among their assigned pixel positions in the generated image. By ensuring that similar features are positioned close to each other, IGTD enhances the ability of CNNs to identify patterns and correlations within the data.

Given a tabular dataset **X**, where $X=\{x_{ij},i=1,\cdots ,n;j=1,\cdots ,p\}$, each row $x_{i}$ represents a sample, and each column *X*_*j*_ corresponds to a feature variable. The objective of tabular-to-image conversion is to transform each row $x_{i}$ into an image of size *N*_*r*_ × *N*_*c*_, where *N*_*r*_ and *N*_*c*_ denote the number of rows and columns in the image, respectively, satisfying the constraint $N_{r}\times N_{c}=p$. We outline the step-by-step procedure of IGTD in the following.

1. Computing the Rank Matrix of Feature Distances, **R**:
  To construct the rank matrix, pairwise distances between features are computed using a selected distance measure, such as the Euclidean distance. These pairwise distances are then ranked in ascending order, ensuring that smaller distances are assigned smaller ranks while larger distances receive larger ranks. The resulting *p* × *p* rank matrix, denoted as **R**, is formed, where each element *r*_*ij*_ at the *i*th row and *j*th column represents the rank of the distance between the *i*th and *j*th features. To maintain consistency, the diagonal elements of **R** are set to zero.
2. Computing the Rank Matrix of Pixel Distances, **Q**:
  For an image of size *N*_*r*_ × *N*_*c*_, the pairwise distances between pixels are computed based on their pixel coordinates, using a chosen distance measure such as the Euclidean distance. These pairwise pixel distances are then ranked in ascending order, ensuring that smaller distances receive smaller ranks while larger distances are assigned larger ranks. The resulting *p* × *p* rank matrix of pixel distances, denoted as **Q**, is constructed, where each element *q*_*ij*_ represents the rank of the distance between pixel *i* and pixel *j*. To maintain consistency, the main diagonal of **Q** is set to zero, and **Q** is ensured to be symmetric. The ordering of pixels in **Q** follows a row-wise concatenation, where pixels in the image are sequentially arranged row by row.
3. Optimizing Feature Arrangement by Minimizing the Difference Between **R** and **Q**:
  To achieve an optimal feature-to-pixel mapping, the rows and columns of **R** are permuted synchronously to minimize the squared difference between **R** and **Q**. The optimization process seeks to minimize the following error function:
  $$err\left(R,Q\right)=\sum_{i=2}^{p}\sum_{j=1}^{i-1}(r_{ij}-q_{ij}{)}^{2},$$
  where err(R,Q) represents the total squared error between the two rank matrices. By iteratively searching for suitable feature swaps and reordering the rows and columns of **R**, the difference between the two matrices is minimized. The optimized feature distance ranking matrix after this process is denoted as $R^{\prime }$.
4. Generating Pseudo-images for Each Sample:
  After obtaining the optimized feature distance ranking matrix $R^{\prime }$, each *i*th feature (corresponding to the *i*th row and column in $R^{\prime }$) is assigned to the *i*th pixel (the *i*th row and column in **Q**). Using these assigned feature locations within the *N*_*r*_ × *N*_*c*_ pixel matrix, *n* pseudo-images are generated, where each image corresponds to a sample in the dataset. This process ensures that the spatial arrangement of features in the pseudo-images preserves meaningful relationships, allowing CNNs to effectively learn patterns from the transformed data.

IGTD has demonstrated substantial versatility across diverse application domains. Nazarri, Yusof, and Almohammedi (2023) [40] applied IGTD to cybersecurity by analyzing real-world network traffic datasets. By transforming these datasets into images and utilizing CNN models for classification, they achieved approximately 80% accuracy in intrusion detection, highlighting IGTD’s potential in identifying cyber threats. Similarly, Hosseini and Chitsaz (2023) [41] leveraged IGTD in the automotive industry to assess the knock probability of turbocharged gasoline engines under real driving conditions. Converting the collected datasets into images and processing them with CNNs resulted in a knock detection accuracy of 89.3%, demonstrating IGTD’s effectiveness in engine diagnostics and performance monitoring.

## IGTD based on non-Euclidean metric

IGTD employs the Euclidean metric to measure dissimilarities between features. Euclidean distance is sensitive to absolute differences and assumes an isotropic space. It is most appropriate when features are independent and have equal variance. It is widely used in clustering tasks and in image retrieval systems where features are raw pixel intensities. Another potential limitation of Euclidean distance is its inherent assumption that features are continuous and linearly related, which may not always hold, particularly in cases involving nonlinear, high-dimensional, or unevenly distributed features. To address these limitations, this study explores alternative distance metrics, one minus correlation (RD), Geodesic distance (GD), Jensen-Shannon distance (JD), Wasserstein distance (WD), and Tropical distance (TD), which may offer more suitable representations under specific conditions. In the following, we provide a detailed discussion of these four distance metrics and their applicability to tabular-to-image conversion in IGTD.

It is important to note that the proposed non-Euclidean metrics are exclusively applied to the computation of the Feature Distance Rank Matrix (R), which captures the dissimilarity between feature vectors in the original tabular space. The Pixel Distance Rank Matrix (Q) continues to be calculated using the Euclidean distance, as the target representation is a fixed two-dimensional image grid where spatial proximity is inherently Euclidean. Consequently, the optimization process seeks to map the intrinsic non-Euclidean relationships captured in R onto the Euclidean layout of Q.

### One minus correlation coefficient as a distance (RD)

The one minus correlation coefficient is a widely used distance metric in bioinformatics, particularly for measuring gene expression similarity [42]. Derived from the Pearson correlation coefficient, which quantifies the linear relationship between two variables, say *X* and *Y*, it transforms correlation into a dissimilarity measure as $D_{R}(X,Y)=1-\rho (X,Y)$, ensuring larger values indicate greater feature dissimilarity.

A key advantage of one minus correlation coefficient is its scale invariance, making it ideal for biological datasets where gene expression levels vary. It remains robust to differences in magnitude, making it useful for clustering and classification by effectively capturing linear dependencies [43]. Additionally, it is computationally efficient, allowing fast calculations in large datasets. Beyond bioinformatics, one minus correlation coefficient is applied in time-series analysis, machine learning, and clustering, playing a critical role in pattern recognition and feature analysis.

Despite its benefits, one minus correlation coefficient has limitations. It only captures linear relationships, making it ineffective for nonlinear dependencies. It is sensitive to outliers, which can distort similarity measures and introduce bias. Moreover, it ignores absolute differences in magnitude, which is problematic in fields like medical diagnostics and financial risk analysis, where similar trends but vastly different values must be distinguished. Additionally, one minus correlation coefficient is not a true distance metric, as it fails the triangle inequality, limiting its use in certain distance-based algorithms and clustering methods.

### Geodesic distance (GD)

Euclidean distance often misrepresents true proximity when data lies on a curved or nonlinear manifold, as it assumes a flat, linear space. In contrast, Geodesic distance accounts for the manifold’s intrinsic geometry by measuring the shortest path along its surface between two points. This makes it especially effective in capturing meaningful relationships within data embedded in complex, curved spaces. By respecting the underlying structure of the space, Geodesic distance offers a more accurate and informative measure of similarity than traditional linear metrics. However, a key limitation of Geodesic distance is that it does not satisfy the triangle inequality, making direct computation infeasible. To overcome this issue, approximation techniques such as the ISOMAP algorithm [44] are employed.

The ISOMAP algorithm combines the computational efficiency of Multidimensional Scaling (MDS) and Principal Component Analysis (PCA) with global optimization and asymptotic convergence to estimate Geodesic distances. It constructs a neighborhood graph by connecting nearby points in high-dimensional space, effectively capturing the local structure of the nonlinear space. The estimated Geodesic distances between feature variables in a tabular dataset **X** are computed as follows:

1. Compute the Euclidean distance *d*_*X*_(*i*,*j*) between every pair of feature variables *X*_*i*_ and *X*_*j*_, where i,j∈{1,…,p}.
2. Construct a graph *G* using the feature variables as nodes. Connect neighboring features based on their distances. If *X*_*i*_ and *X*_*j*_ are neighbors, set
  $$d_{G}(i,j)=d_{X}(i,j).$$
  Otherwise, assign
  $$d_{G}(i,j)=\infty$$
  to indicate no direct connection between the two features.
3. Update *d*_*G*_(*i*,*j*) iteratively using the equation:
  $$d_{G}(i,j)=\min\{d_{G}(i,j),d_{G}(i,k)+d_{G}(k,j)\},\;\text{for }k=1,\ldots ,p.$$
  The resulting matrix $D_{G}=\{d_{G}(i,j)\}$ represents the minimum distances and provides an estimate of the Geodesic distance among feature variables.

In this study, the neighborhood graph was constructed using a *k*-nearest neighbor method. To mitigate topological instability, often referred to as the “pinch problem” where the graph becomes disconnected, we adopted a single-linkage strategy where the distance between disconnected components was approximated by the minimum Euclidean distance between their closest points. By leveraging the ISOMAP algorithm, Geodesic distances can be effectively estimated, allowing for a more accurate representation of relationships within nonlinear feature spaces. This method enhances the ability of CNNs to capture complex feature structures when transforming tabular data into image representations.

### Jensen-Shannon distance (JD)

The Jensen-Shannon (JS) distance [45] is a statistical measure used to quantify the similarity between two probability distributions. It is based on the Jensen-Shannon divergence, which, unlike the Kullback-Leibler (KL) divergence, is both symmetric and satisfies the triangle inequality, qualifying it as a true metric. The JS distance between two distributions *X* and *Y* is defined as:


$$D_{JS}(X\;\|\;Y)=\sqrt{\frac{1}{2}KL(X\;\|\;M)+\frac{1}{2}KL(Y\;\|\;M)},$$

where M=(X+Y)/2 and *KL* denotes the Kullback-Leibler divergence.

This distance ranges from 0 to 1, where 0 indicates identical distributions and 1 indicates completely different distributions. JS distance is sensitive to both the shape and magnitude of the input distributions. Unlike Euclidean distance, which assumes feature homogeneity and fails to account for probabilistic structure, JS distance excels at comparing normalized distributions, such as frequency profiles or categorical probabilities. For high-dimensional feature spaces where variables may not follow a common distribution, JS distance offers a robust alternative to traditional metrics. It effectively captures the local structure and relationships among features by comparing the underlying probability distributions, making it especially useful when the data exhibits non-uniform or categorical characteristics.

In practice, JS distance has been widely used in fields like natural language processing, particularly for topic modeling and document classification [46], and in single-cell RNA sequencing, where it aids in comparing cell-type-specific gene expression distributions [47]. Its robustness, boundedness, and interpretability make it a valuable tool for analyzing structured, probability-based data.

To apply the Jensen-Shannon distance to continuous tabular data, such as gene expression profiles, we treat each feature vector as a discrete Probability Mass Function (PMF) defined over the sample space. Specifically, let $x_{\cdot j}$ represent the vector of values for the *j*-th feature across *n* samples. We normalize this vector to sum to unity:


$$P_{ij}=\frac{x_{ij}}{\sum_{k=1}^{n}x_{kj}}$$

where *P*_*ij*_ represents the probability mass associated with sample *i* for feature *j*. If the data had contained negative values, such as *z*-scores, a Softmax normalization would be applied instead. Unlike a Kernel Density Estimation (KDE) which estimates the distribution of values, this PMF approach preserves the sample-wise structure, allowing the JS distance to quantify the dissimilarity between feature profiles.

### Wasserstein distance (WD)

The Wasserstein distance [48], also referred to as the Earth Mover’s Distance (EMD) or Mallows distance, quantifies the minimal cost required to transform one probability distribution into another by optimally transporting probability mass. Unlike traditional pointwise metrics, it considers both the amount of mass moved and the distance it is moved, making it sensitive to the geometry of distributions. To compute the Wasserstein distance between two continuous distributions *F*_*X*_ and *F*_*Y*_ on a two-dimensional plane, the following steps are taken:

1. For quantile functions $F_{X}^{-1}$ and $F_{Y}^{-1}$, the Wasserstein distance is computed as:
  $$W_{2}(X,Y)=\|F_{X}^{-1}-F_{Y}^{-1}{\|}_{2}={\left(\int_{0}^{1}{\left|F_{X}^{-1}(\alpha )-F_{Y}^{-1}(\alpha )\right|}^{2}d\alpha \right)}^{\frac{1}{2}}.$$
2. Closed-form formulae are only available when *X* and *Y* follow a Gaussian distribution. If $X\sim N(\mu_{X},\sigma_{X}^{2})$ and $Y\sim N(\mu_{Y},\sigma_{Y}^{2})$, then the squared Wasserstein distance is represented as:
  $$W_{2}^{2}(X,Y)=\|F_{X}^{-1}-F_{Y}^{-1}{\|}_{2}^{2}=(\mu_{X}-\mu_{Y}{)}^{2}+(\sigma_{X}-\sigma_{Y}{)}^{2}+2\sigma_{X}\sigma_{Y}(1-\rho_{XY}),$$
  where $\rho_{XY}$ is the Pearson correlation coefficient.

This distance is particularly advantageous for comparing complex, non-uniform distributions in high-dimensional spaces. It captures both shape and location differences between distributions, offering a more nuanced measure than divergence-based metrics like KL or JS divergence. Furthermore, Wasserstein distance is inherently robust to sample variability and outliers, making it suitable for analyzing empirical distributions such as histograms or quantiles.

Due to these properties, Wasserstein distance has become a foundational tool in various machine learning applications. It is central to generative modeling, most notably in Wasserstein GANs [49], where it addresses instability issues in adversarial training. In spatial transcriptomics, it facilitates the comparison of gene expression distributions across spatial regions [50]. Additionally, it has proven effective in tasks like histogram-based image classification, where it accurately reflects the underlying structure of the data.

### Tropical distance (TD)

Recent research has highlighted the growing importance of non-Euclidean distance measures in machine learning and data analysis. Among these, the tropical distance, also known as the generalized Hilbert projective metric, is capable of capturing more complex data geometries and hierarchical relationships. The tropical distance originates from the field of tropical geometry [51], in which standard arithmetic operations are replaced by the “min-plus” or “max-plus” algebra. This framework provides a piecewise-linear and combinatorial alternative to classical Euclidean geometry, enabling the modeling of high-dimensional and structured data.

In this study, we define the tropical distance between two feature variables, *X*_*i*_ and *X*_*j*_, as


$$d_{T}(X_{i},X_{j})=\max_{k}(x_{ki}-x_{kj})-\min_{k}(x_{ki}-x_{kj}).$$

The resulting matrix $D_{T}=\{d_{T}(X_{i},X_{j}),i,j\in \{1,\cdots ,p\}\}$ represents the tropical distance among feature variables. The tropical distance measures the range of coordinate-wise differences and reflects the relative structural variations between vectors rather than their absolute magnitudes. Owing to its scale-invariant and polyhedral nature, tropical geometry provides a robust foundation for representing complex relationships among multivariate data.

This approach has demonstrated significant potential in advancing neural network capabilities. For instance, Yoshida, Aliatimis, and Miura (2024) [52] proposed tropical neural networks, which have been successfully applied to the classification of complex and structured data such as phylogenetic trees, illustrating their effectiveness in capturing hierarchical patterns. Furthermore, Pasque et al. (2024) [53] introduced tropical decision boundaries that have been shown to provide notable robustness against adversarial attacks, revealing an inherent stability often lacking in Euclidean-based models. These advances collectively highlight the promise of tropical and other non-Euclidean metrics in enhancing data representation, model robustness, and classification performance within modern computational frameworks. Motivated by these findings, we integrate the tropical distance into our comparative analysis of non-Euclidean metrics within the IGTD framework for converting tabular data into images, aiming to evaluate its potential for improving both the performance and resilience of subsequent CNN-based classification models.

## Simulation studies

To evaluate the ability of various distance metrics to capture complex relationships among features in high-dimensional settings, we conducted a series of simulation studies. Specifically, we generated six independent datasets, each intentionally constructed to align with one of the following distance metrics: Euclidean distance, one minus correlation, Geodesic distance, Jensen-Shannon distance, and Wasserstein distance. Each dataset comprises *n* = 200 observations and *p* = 2500 predictors. Let the data be denoted as $\left\{Y_{i},X_{i1},X_{i2},\ldots ,X_{ip}\right\}$, where $Y_{i}\in \{0,1\}$ is a binary response variable for the *i*-th observation, and $X_{ij}\in \mathbb{R}$ represents the value of the *j*-th predictor for the *i*-th observation.

In every dataset, the first 100 predictors $\left(X_{i1},\ldots ,X_{i100}\right)$ are structured according to the geometric or distributional properties best captured by the corresponding distance metric. In all scenarios, the remaining predictors $X_{i101},\ldots ,X_{i2500}$ are added as standard normal noise to replicate the challenges of high-dimensional settings commonly found in real-world applications. The simulation design ensures that the primary signal is confined to the first 100 features and is distinctly structured to favor a particular distance metric. This simulation framework allows for a systematic evaluation of the ability of each distance metric to capture meaningful relationships in data with diverse geometrical and distributional characteristics. The results offer insights into the applicability and limitations of distance-based modeling approaches in high-dimensional structured data environments. The specific construction of each dataset is described in detail as follows.

### Euclidean distance-based model

In this setting, the first 100 predictors are simulated from a multivariate normal distribution with independent and identically distributed components, capturing a linear structure well-suited to Euclidean geometry. The binary response variable $Y_{i}\in \{0,1\}$ is generated using a logistic regression model, where the linear predictor is formed from these signal features. Euclidean distance is particularly effective when data resides in a flat, linear space with uncorrelated and equally scaled features. Under this model, the predictors follow a multivariate normal distribution with identity covariance, naturally inducing a Euclidean geometry.

Let the signal predictors be denoted by $X_{i}^{\left(E\right)}=(X_{i1},X_{i2},\ldots ,X_{i100})$ $\in {\mathbb{R}}^{100}$, where each component is independently drawn from a standard normal distribution. The model is specified as follows:


$$X_{i}^{\left(E\right)}\sim 𝒩(0,2I_{100}),\;\beta^{\left(E\right)}\sim 𝒩(2.5,I_{100}),$$

$$\text{logit}(P(Y_{i}=1))=X_{i}^{\left(E\right)}\cdot \beta^{\left(E\right)}.$$

The binary response *Y*_*i*_ is generated from a Bernoulli distribution with probability *P*(*Y*_*i*_ = 1). This formulation assumes that the similarity between observations is best captured by their Euclidean proximity in the high-dimensional predictor space. We purposely maintained a high noise-to-signal ratio (2400 noise features versus 100 signal features) in this setting. This parameter choice was intended to serve as a stress test for the curse of dimensionality, evaluating whether the distance metrics could recover a linear signal buried in substantial noise without prior feature selection.

### One minus correlation-based model

To construct a dataset where the similarity among observations is best captured by one minus correlation distance, the first 100 predictors are generated from a multivariate normal distribution with a strong Toeplitz correlation structure: $\Sigma_{jk}=\rho^{\left|j-k\right|}$, where ρ=0.95. This structure ensures high linear dependencies among features, which enhances the sensitivity of correlation-based metrics. Unlike random coefficients, we design the coefficient vector $\beta^{\left(C\right)}$ to follow a smooth monotonic trend, increasing gradually across dimensions, which helps align the discriminative signal with the correlation structure.

Let $X_{i}^{\left(C\right)}=(X_{i1},\ldots ,X_{i100})\in {\mathbb{R}}^{100}$ represent the signal features drawn from:


$$X_{i}^{\left(C\right)}\sim 𝒩(0,\Sigma^{\left(C\right)}),\;\text{where}\;\Sigma_{jk}^{\left(C\right)}=\rho^{\left|j-k\right|},\;\rho =0.95.$$

We define a structured coefficient vector:


$$\beta^{\left(C\right)}=\left(\frac{1}{100},\frac{2}{100},\ldots ,1\right),$$

which distributes signal smoothly across features in a correlated fashion.

The linear predictor is scaled to amplify the signal:


$$\text{logit}(P(Y_{i}=1))=3\cdot X_{i}^{\left(C\right)}\cdot \beta^{\left(C\right)}.$$

Finally, the binary response is generated from a Bernoulli distribution using the logistic probabilities. The remaining 2400 predictors are added as standard Gaussian noise, resulting in a high-dimensional dataset with 2500 total features. This formulation ensures that class-discriminative information is embedded in the linear correlation patterns, favoring the one minus correlation distance (IGTD(RD)) as the most appropriate similarity measure.

### Geodesic distance-based model

The signal features are sampled from a nonlinear manifold, such as a Swiss roll, embedded in high-dimensional space. The binary response *Y*_*i*_ is determined based on the Geodesic distance along the manifold, reflecting intrinsic, non-Euclidean feature relationships. Let $X_{i}^{\left(G\right)}=(X_{i1},\ldots ,X_{i100})\in {\mathbb{R}}^{100}$ lie on a low-dimensional nonlinear manifold $ℳ\subset {\mathbb{R}}^{100}$, e.g., a Swiss roll:


$$X_{i}^{\left(G\right)}\in ℳ,\;\text{dim}(ℳ)\ll 100$$

Let $f:{\mathbb{R}}^{100}\to \mathbb{R}$ be a nonlinear function defined over the Geodesic distance from a reference point, the binary response *Y*_*i*_ is obtained by


$$Y_{i}=𝕀\left(f(X_{i}^{\left(G\right)})>\tau \right),$$

where τ is the median of $\left\{f(X_{i}^{\left(G\right)})\right\}$.

### Jensen-Shannon distance-based model

In this model, the first 100 predictors represent compositional or probability-valued features, drawn from class-specific Dirichlet distributions. This setup is designed to favor the Jensen-Shannon (JS) distance, which effectively captures differences between discrete probability distributions. For each observation *i*, the binary response variable $Y_{i}\in \{0,1\}$ determines the distribution from which the signal features are generated. Let $X_{i}^{\left(JS\right)}=(X_{i1},\ldots ,X_{i100})\in \Delta^{99}$ denote a probability vector lying on the 99-simplex. Then, the class-conditional generation is given by:


$$Y_{i}\sim \text{Bernoulli}(0.5),$$

$$X_{i}^{\left(JS\right)}|Y_{i}=0\sim \text{Dirichlet}(\alpha_{0}),\;\alpha_{0}=(0.1,\ldots ,0.1),$$

$$X_{i}^{\left(JS\right)}|Y_{i}=1\sim \text{Dirichlet}(\alpha_{1}),\;\alpha_{1}=(5,\ldots ,5).$$

Here, $\alpha_{0}$ and $\alpha_{1}$ are chosen to generate distinct yet plausible class-specific distributions: $\alpha_{0}$ produces a uniform distribution, while $\alpha_{1}$ concentrates the probability mass more evenly, resulting in lower entropy. This construction induces differences in the distributional structure of the features, such that the JS distance between observations from different classes is maximized in expectation. The remaining 2400 predictors are standard Gaussian noise, completing the high-dimensional structure. This simulation design ensures that class separation is primarily encoded in distributional divergence, favoring the Jensen-Shannon distance as an appropriate similarity measure.

### Wasserstein distance-based model

To construct a setting where the Wasserstein distance effectively captures class differences, the signal features are simulated as empirical quantile vectors derived from class-specific distributions. Specifically, for each observation *i*, the signal vector $X_{i}^{\left(W\right)}=(X_{i1},\ldots ,X_{i100})\in {\mathbb{R}}^{100}$ is generated by sorting a sample of 100 values drawn from an underlying distribution depending on the class label $Y_{i}^{\prime }\in \{0,1\}$. The class-conditional distributions are defined as follows:


$$Y_{i}^{\prime }\sim \text{Bernoulli}(0.5),$$

$$X_{i}^{\left(W\right)}|Y_{i}^{\prime }=0\sim \text{Sort}\left({\text{Sample}}_{100}\left(𝒩(0,1)\right)\right),$$

$$X_{i}^{\left(W\right)}|Y_{i}^{\prime }=1\sim \text{Sort}\left({\text{Sample}}_{100}\left(𝒩(1,1)\right)\right).$$

Let $T\in {\mathbb{R}}^{100}$ be a fixed template distribution, defined as the average quantile vector computed from all class 0 observations. The Wasserstein distance is computed as:


$$W_{1}(X_{i}^{\left(W\right)},T)=\sum_{j=1}^{100}\left|\sum_{k=1}^{j}(X_{ik}-T_{k})\right|.$$

The binary class label is then determined by:


$$Y_{i}=𝕀\left(W_{1}(X_{i}^{\left(W\right)},T)>\tau \right),$$

where τ is the empirical median of the Wasserstein distances across all observations. This construction ensures that class separation is encoded via both location and shape differences in the empirical distributions, making Wasserstein distance the most suitable similarity measure for classification in this setting.

Although the Wasserstein distance is theoretically capable of capturing differences in both distribution shape (e.g., variance, skewness) and location, this simulation focuses exclusively on a location shift between class-conditional distributions. This design choice was made to isolate the location effect and provide a controlled evaluation of the metric’s sensitivity to mean differences, ensuring that the source of the discriminative signal is unambiguous.

### Tropical distance-based model

To construct a simulation setting in which the tropical distance is uniquely aligned with the intrinsic discriminative structure, we design a feature space whose geometry is governed by coordinate-wise dominance patterns rather than Euclidean magnitude, linear correlation, or smooth manifold structure. The tropical distance between two vectors $x,y\in {\mathbb{R}}^{n}$ is defined as


$$d_{T}(x,y)=\max_{k}(x_{k}-y_{k})-\min_{k}(x_{k}-y_{k}),$$

which measures the range of coordinate-wise differences and is invariant to additive shifts. This metric is therefore particularly sensitive to max-min dominance relations.

In this model, the first 100 predictors encode the tropical signal, while the remaining *p*–100 predictors act as independent noise. Let $X_{i}^{\left(T\right)}=(X_{i1}^{\left(T\right)},\ldots ,X_{i,100}^{\left(T\right)})\in {\mathbb{R}}^{100}$ denote the signal features for the *i*th observation and $Y_{i}\in \{0,1\}$ the corresponding binary class label, with $Y_{i}\sim Bernoulli(0.5)$ independently for i=1,…,n. The index set {1,…,100} is randomly partitioned into *S* disjoint segments $\left\{I_{1},\ldots ,I_{S}\right\}$ of approximately equal size, each representing a plateau of nearly constant feature values. For each observation *i* and segment s∈{1,…,S}, we first draw an independent baseline level


$$v_{is}\sim Uniform(-1,1).$$

To introduce global class-dependent structure, two distinct segments *s*^ + ^ and *s*^−^ are selected once per dataset, uniformly from {1,…,S} with $s^{+}\neq s^{-}$. These segments are shifted upward and downward, respectively, for all observations in class 1, while class 0 observations retain only the baseline levels:


$$v_{is}^{\left(Y_{i}\right)}=\left\{ \begin{array}{cc} v_{is}, & \text{if }Y_{i}=0,\\ v_{is}+\Delta \cdot 𝕀(s=s^{+})-\Delta \cdot 𝕀(s=s^{-}), & \text{if }Y_{i}=1, \end{array}\right.$$

where Δ>0 controls the strength of the tropical signal. To avoid degeneracy and introduce mild within-segment variability, we add a small Gaussian perturbation and set


$$X_{ij}^{\left(T\right)}=v_{is}^{\left(Y_{i}\right)}+\varepsilon_{ij},\;\varepsilon_{ij}\sim 𝒩(0,\sigma_{seg}^{2}),\;j\in I_{s},\;i=1,\ldots ,n.$$

Unless otherwise stated, we set *S* = 5, choose Δ∈[1.5,2.0], and use $\sigma_{seg}=0.10$.

After simulating the 100 signal features, we optionally apply column-wise centering across samples,


$$X_{ij}^{\left(T\right)}\leftarrow X_{ij}^{\left(T\right)}-\frac{1}{n}\sum_{k=1}^{n}X_{kj}^{\left(T\right)},$$

which removes mean differences between feature vectors. This operation alters Euclidean and correlation-based distances between features but leaves the tropical distance *d*_*T*_ unchanged, because subtracting a constant from both vectors does not affect the range of their coordinate-wise differences. Thus, column centering suppresses some of the geometric cues preferred by Euclidean and correlation metrics while preserving the dominance structure emphasized by the tropical distance. The remaining *p*–100 predictors are generated as independent Gaussian noise:


$$Z_{i}\sim 𝒩(0,\sigma_{n}^{2}I_{p-100}),\;\sigma_{n}=0.01,$$

and the full predictor vector is defined as


$$X_{i}=(X_{i}^{\left(T\right)},Z_{i})\in {\mathbb{R}}^{p}.$$

From a geometric perspective, this construction induces a clear block structure in the tropical distance matrix between features. For any two feature vectors belonging to the same segment *I*_*s*_, the coordinate-wise differences across samples remain small and nearly constant, yielding a small tropical distance *d*_*T*_. In contrast, pairs of features from different segments, and especially those between the globally shifted segments $I_{s^{+}}$ and $I_{s^{-}}$, exhibit large and systematic coordinate-wise dominance gaps, resulting in substantially larger tropical distances. Consequently, when IGTD is built using the tropical distance, the resulting feature distance rank matrix closely approximates a block-structured ideal, and the optimized pixel arrangement places segment features into coherent spatial clusters on the image grid.

By comparison, Euclidean and one minus correlation distances primarily depend on differences in global means, variances, or linear dependencies, which are moderated by the symmetric Uniform(−1,1) baseline and further reduced by column-wise centering. Geodesic, Jensen-Shannon, and Wasserstein distances rely on smooth manifold geometry or fine-grained distributional form, both of which are weakened by the piecewise-constant plateau construction and the addition of isotropic Gaussian noise. As a result, these non-tropical metrics fail to reveal the strong segment-wise geometry that determines the class boundary, whereas the tropical distance, being explicitly sensitive to coordinate-wise dominance and invariant to translation, remains uniquely aligned with the true discriminative structure. Within the IGTD framework, this alignment enables IGTD(TD) to generate pseudo-images with distinct, localized intensity patterns that are strongly associated with the class label, allowing CNN-based classifiers to achieve substantially higher accuracy than when IGTD is built on Euclidean, correlation-based, Geodesic, Jensen-Shannon, or Wasserstein distances.

### Results

**Fig 1:**
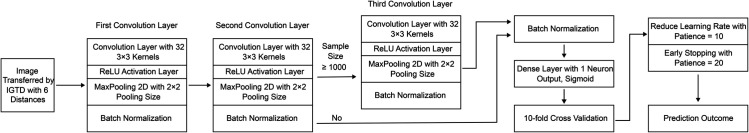
Schematic of the CNN architecture used for classification. The network comprises two sequential blocks, each containing a convolutional layer with 3 × 3 kernels (stride 1, same padding), batch normalization, and a max-pooling layer. The final layers are tailored for binary classification, utilizing binary cross-entropy loss. Training was optimized with a learning rate reduction strategy and early stopping.

The generated datasets were processed using the IGTD algorithm to produce image representations based on six different distance metrics. Note that the computation of pixel distances in the IGTD algorithm is always based on Euclidean distances. These images were then input into a standardized, pre-trained convolutional neural network (CNN). The CNN architecture comprised two convolutional layers with 3 × 3 kernels, followed by max-pooling layers and batch normalization. We used a stride of 1 and same padding (zero padding to preserve spatial dimensions) for the convolutional layers. Binary cross-entropy was used as the loss function. Model training and evaluation were performed using ten-fold cross-validation repeated across ten iterations. A learning rate reduction strategy and early stopping mechanism were employed to optimize training efficiency and prevent overfitting. The full architecture of the CNN is illustrated in Fig 1.

To assess classification performance, we used standard validations indices (VIs) including accuracy, precision, recall, and F1-score. The performance of the IGTD algorithm using non-Euclidean distance metrics was compared against a baseline model that employed the Euclidean distance. Specifically, we denote IGTD(ED) for the Euclidean distance, IGTD(RD) for the one minus correlation distance, IGTD(GD) for the Geodesic distance, IGTD(JS) for the Jensen-Shannon distance, IGTD(WD) for the Wasserstein distance, and IGTD(TD) for the Tropical distance. Ten-fold cross-validation classification results (mean and standard deviation) across six simulated model datasets, using six different distance metrics within the IGTD framework are shown in Table 1. The classification results across the six simulated models demonstrate that each distance metric within the IGTD framework performs best under specific data structures that align with its mathematical properties.

**Table 1:** Ten-fold cross-validation classification results (mean and standard deviation) across six simulated model datasets, using six different distance metrics within the IGTD framework.

(a) Euclidean distance-based model						
VIs	IGTD(ED)	IGTD(RD)	IGTD(GD)	IGTD(JD)	IGTD(WD)	IGTD(TD)
Accuracy	0.5200 (0.0714)	0.5150 (0.0896)	0.5150 (0.1141)	0.4550 (0.0757)	0.4950 (0.1083)	0.5100 (0.0889)
Precision	0.5512 (0.1682)	0.5076 (0.0761)	0.4767 (0.2120)	0.4179 (0.1225)	0.5394 (0.1737)	0.4698 (0.1904)
Recall	0.4811 (0.2559)	0.6289 (0.2132)	0.4511 (0.2418)	0.3322 (0.1893)	0.4578 (0.1753)	0.5567 (0.2453)
F1-Score	0.4577 (0.1419)	0.5384 (0.1115)	0.4446 (0.1971)	0.3462 (0.1403)	0.4487 (0.1131)	0.4934 (0.1791)
**(b) One minus correlation-based model**						
VIs	IGTD(ED)	IGTD(RD)	IGTD(GD)	IGTD(JD)	IGTD(WD)	IGTD(TD)
Accuracy	0.6100 (0.1530)	0.7800 (0.1187)	0.6450 (0.0610)	0.7200 (0.1345)	0.5950 (0.1457)	0.6950 (0.1059)
Precision	0.5923 (0.1542)	0.7897 (0.1561)	0.6293 (0.0747)	0.7471 (0.1524)	0.5820 (0.2580)	0.7309 (0.1403)
Recall	0.6744 (0.2450)	0.7967 (0.1258)	0.7122 (0.1142)	0.7422 (0.2192)	0.5867 (0.3345)	0.7389 (0.2531)
F1-Score	0.6154 (0.1794)	0.7823 (0.1068)	0.6614 (0.0557)	0.7105 (0.1594)	0.5328 (0.2615)	0.6789 (0.1790)
**(c) Geodesic distance-based model**						
VIs	IGTD(ED)	IGTD(RD)	IGTD(GD)	IGTD(JD)	IGTD(WD)	IGTD(TD)
Accuracy	0.7700 (0.0927)	0.7950 (0.1011)	0.8700 (0.0557)	0.8200 (0.1030)	0.8600 (0.0800)	0.7450 (0.1404)
Precision	0.7728 (0.1126)	0.8257 (0.1148)	0.8377 (0.0765)	0.8255 (0.1288)	0.8698 (0.1188)	0.7295 (0.1885)
Recall	0.8000 (0.0894)	0.7800 (0.2088)	0.9300 (0.0781)	0.8300 (0.1005)	0.8700 (0.0458)	0.7300 (0.2147)
F1-Score	0.7796 (0.0747)	0.7798 (0.1281)	0.8778 (0.0524)	0.8237 (0.0982)	0.8654 (0.0680)	0.7263 (0.2023)
**(d) Jensen-Shannon distance-based model**						
VIs	IGTD(ED)	IGTD(RD)	IGTD(GD)	IGTD(JD)	IGTD(WD)	IGTD(TD)
Accuracy	1.0000 (0.0000)	0.5700 (0.0980)	0.9850 (0.0229)	0.9900 (0.0200)	1.0000 (0.0000)	0.8300 (0.1568)
Precision	1.0000 (0.0000)	0.3567 (0.3067)	0.9709 (0.0444)	0.9809(0.0382)	1.0000 (0.0000)	0.8316 (0.1732)
Recall	1.0000 (0.0000)	0.3156 (0.2911)	1.0000 (0.0000)	1.0000 (0.0000)	1.0000 (0.0000)	0.8111 (0.2138)
F1-Score	1.0000 (0.0000)	0.3327 (0.2973)	0.9847 (0.0234)	0.9900 (0.0201)	1.0000 (0.0000)	0.8137 (0.1814)
**(e) Wasserstein distance-based model**						
VIs	IGTD(ED)	IGTD(RD)	IGTD(GD)	IGTD(JD)	IGTD(WD)	IGTD(TD)
Accuracy	0.9850 (0.0229)	0.9850 (0.0229)	0.9800 (0.0332)	0.9850 (0.0229)	0.9900 (0.0200)	0.9900 (0.0200)
Precision	1.0000 (0.0000)	1.0000 (0.0000)	0.9900 (0.0230)	1.0000 (0.0000)	0.9900 (0.0200)	1.0000 (0.0000)
Recall	0.9700 (0.0458)	0.9700 (0.0458)	0.9700 (0.0458)	0.9700 (0.0458)	1.0000 (0.0000)	0.9800 (0.0400)
F1-Score	0.9842 (0.0241)	0.9842 (0.0241)	0.9795 (0.0337)	0.9842 (0.0241)	0.9949 (0.0103)	0.9895 (0.0211)
**(f) Tropical distance-based model**						
VIs	IGTD(ED)	IGTD(RD)	IGTD(GD)	IGTD(JD)	IGTD(WD)	IGTD(TD)
Accuracy	0.8050 (0.1106)	0.9150 (0.0594)	0.8950 (0.0568)	0.8500 (0.0707)	0.8500 (0.0671)	0.9150 (0.0550)
Precision	0.8121 (0.1377)	0.8984 (0.0779)	0.9407 (0.0937)	0.8958 (0.0977)	0.9050 (0.0827)	0.9457 (0.0714)
Recall	0.7300 (0.2309)	0.9233 (0.0707)	0.8400 (0.1089)	0.7856 (0.1384)	0.7644 (0.1320)	0.8722 (0.0934)
F1-Score	0.7534 (0.2050)	0.9091 (0.0646)	0.8800 (0.0670)	0.8252 (0.0947)	0.8213 (0.0876)	0.9041 (0.0658)

In the Euclidean distance-based model, IGTD(ED) yielded the highest mean accuracy (0.52) relative to the non-Euclidean alternatives, although the absolute performance was low across all methods. This is attributed to two factors: the high-dimensional noise (2400 noise features vs. 100 signal features) and the use of a standardized CNN architecture. To ensure a fair comparison across all simulation scenarios, we did not fine-tune the CNN hyperparameters specifically for the Euclidean model. Consequently, these results serve as a baseline validation: they demonstrate that under strict “ceteris paribus” conditions, utilizing more complex non-Euclidean metrics offers no predictive benefit when the underlying data structure is strictly linear and i.i.d. Thus, while the challenging experimental conditions dampened the absolute classification power, the relative performance confirms that Euclidean distance remains the most appropriate and parsimonious metric when the data lacks intrinsic non-linear manifolds.

For the correlation-based model, IGTD(RD) emerges as the strongest performer with an accuracy of 0.78, precision of 0.79, and F1-score of 0.78. This reflects the model’s deliberate design: the use of Toeplitz covariance and a gradually increasing coefficient vector emphasizes linear dependency across features, which is effectively captured by the one minus correlation distance. Other metrics, particularly IGTD(JD) and IGTD(WD), show lower and more variable performance, indicating they are not well-suited for such correlated structures.

In the Geodesic distance-based model, IGTD(GD) significantly outperforms all other metrics, with an accuracy of 0.87 and F1-score of 0.88. This aligns well with the underlying data, which are sampled from a nonlinear manifold. The strength of IGTD(GD) lies in its ability to preserve local geometry, making it particularly effective for data residing on curved or non-Euclidean spaces. Metrics like IGTD(ED) and IGTD(RD) are less capable of capturing this structure and thus perform moderately.

Under the Jensen-Shannon model, IGTD(JD) shows excellent performance with an accuracy of 0.99 and F1-score of 0.99, though IGTD(ED) and IGTD(WD) still achieve perfect accuracy in this setting. While this suggests that several metrics can handle compositional features well, IGTD(JD) remains the most interpretable and theoretically appropriate choice for data represented by probability vectors. The fact that all metrics perform strongly here might be due to the pronounced class differences introduced via the Dirichlet parameters.

In the Wasserstein distance-based model, IGTD(WD) performs best overall, achieving the highest accuracy (0.99) and F1-score (0.99), with perfect recall. This is consistent with the model’s design, which encodes class differences through quantile-based distributional shifts. The Wasserstein distance’s sensitivity to both location and shape differences in distributions makes it particularly powerful in this context. Other metrics also perform well, but not as consistently or robustly.

Finally, in the Tropical distance-based model, IGTD(TD) demonstrated superior performance metrics, achieving the highest precision (0.9457) and F1-score (0.9041), alongside a high accuracy (0.9150) comparable to the Euclidean baseline. This suggests that while Euclidean distance can capture some signal in max-plus algebraic structures due to scaling effects, the Tropical distance provides a more precise characterization of the coordinate-wise dominance relationships inherent in the data generation process. The distinct advantage in precision highlights the specific alignment of IGTD(TD) with the tropical geometry of the features.

In summary, these results affirm that each non-Euclidean distance metric excels under the conditions it was designed to model. While the performance gaps between the proposed non-Euclidean metrics and the Euclidean baseline are not always large in these controlled simulations, the results validate a crucial principle of structural alignment. In every scenario, the distance metric that mathematically corresponds to the underlying generative process (e.g., Geodesic distance for manifold data, Wasserstein for distributional shifts, Tropical distance for max-plus structures) consistently achieved the top-ranking performance. This confirms that the IGTD framework functions as intended: it effectively translates specific geometric and distributional relationships into image representations. The simulation study thus serves as a proof of concept, demonstrating that choosing the correct metric provides a consistent, albeit sometimes marginal, advantage in controlled settings, a benefit that becomes more pronounced in complex, real-world data where multiple structural characteristics may coexist.

## Real data examples

### Datasets

Building on the successful validation through statistical simulations, our study progresses to applying the proposed method to real-world datasets. We utilize the same CNN models employed in the simulation study to maintain consistency in evaluation. Performance assessment is conducted using key evaluation metrics, including accuracy, precision, recall, and F1-score, ensuring a comprehensive comparison of model effectiveness. Below, we provide a summarized description of the genetic datasets used in this study (Table 2).

**Table 2:** The summary of the datasets used in this study.

Dataset	Publication	*n*	*p*	Classes (0, 1)
ARCENE	Guyon et al. (2004)	200	10000	112 control, 88 cancer
Colon	Alon et al. (1999)	62	2000	22 normal, 40 tumor
Leukemia	Golub et al. (1999)	72	3571	25 AML, 47 ALL
Ovarian	Petricoin et al. (2002)	253	15154	91 normal, 162 cancer
Prostate	Singh et al. (2002)	102	6033	50 normal, 52 tumor

**ARCENE dataset.** [54] ARCENE was constructed by merging three mass spectrometry datasets: NCI ovarian cancer data, NCI prostate cancer data, and EVMS prostate cancer data. The dataset includes 100 training samples and 100 validation samples, each containing 10,000 features, of which 7000 are predictive and 3000 are spurious. Since the testing set does not provide binary labels, we combined the training and validation sets, resulting in 112 control samples labeled as class 0 and 88 cancer samples labeled as class 1.

**Colon cancer dataset.** [55] This dataset consists of the 2000 genes with the highest minimal intensity across samples and contains 62 samples, including 22 normal and 40 tumor tissues. The binary target variable *y* is set to class 0 for normal tissues and class 1 for tumor tissues.

**Leukemia dataset.** [56] This dataset differentiates between acute myeloid leukemia (AML) and acute lymphoblastic leukemia (ALL). It comprises 72 samples, with 25 AML cases and 47 ALL cases, and includes 3571 gene expression features. The binary target variable *y* is defined as class 0 for AML and class 1 for ALL.

**Ovarian cancer dataset.** [57] This dataset consists of 253 samples, including 91 normal ovarian tissue samples and 162 ovarian cancer samples, with 15,154 molecular mass/charge (M/Z) features. The binary target variable *y* is labeled as class 0 for normal samples and class 1 for cancer samples.

**Prostate cancer dataset.** [58] This dataset contains prostate tissue samples from 102 patients, comprising 52 tumor samples and 50 non-tumor samples, with 6033 gene expression features. The binary target variable *y* is defined as class 0 for non-tumor samples and class 1 for tumor samples.

### Results of the feature distance rank matrices

**Fig 2:**
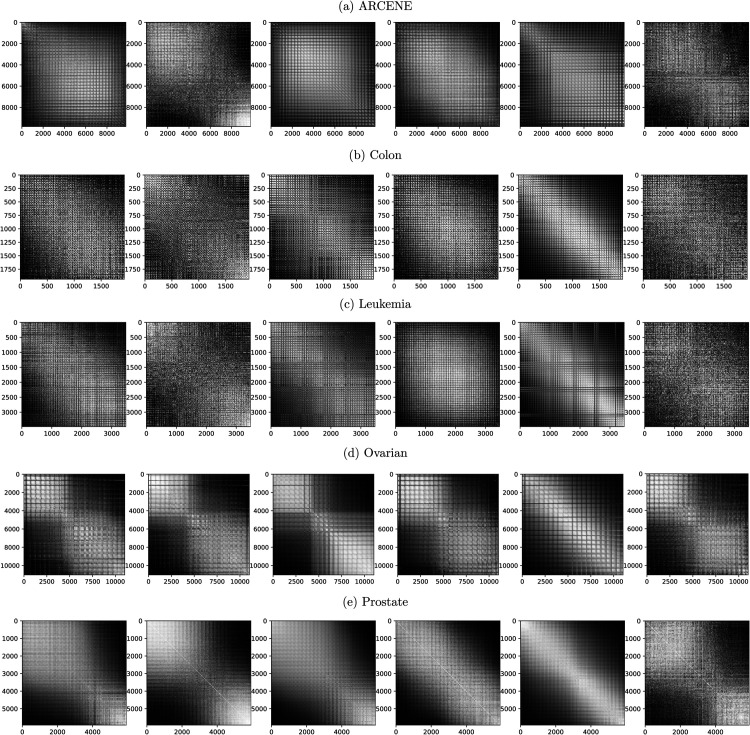
Feature distance rank matrices across different metrics. Feature distance rank matrices based on various distance metrics (from left to right: Euclidean distance, one minus correlation, Geodesic distance, Jensen-Shannon distance, Wasserstein distance, and Tropical distance) computed between all pairs of variables across five datasets, following optimization and feature reordering. The grey level indicates the rank value.

To evaluate the effectiveness of different distance metrics in capturing spatial and structural relationships among features, we compare feature distance rank matrices derived from five real-world tabular datasets against a theoretical “true model.” This reference model represents the rank matrix of Euclidean distances between all pixel pairs in a *p* × *p* image grid, arranged row-wise. Its rank values, visualized through grayscale intensities, exhibit a smooth, symmetric structure, with lighter tones near the diagonal and darker shades radiating outward, reflecting spatial continuity and locality in a well-ordered 2D layout. Fig 2 presents the rank matrices obtained using six distance metrics, Euclidean, one minus correlation, Geodesic, Jensen-Shannon, Wasserstein, and Tropical after feature reordering and optimization to approximate this ideal structure.

Across datasets, Euclidean-based matrices show moderate spatial coherence. For instance, in Prostate, they retain some diagonal structure and smooth transitions. However, in smaller or noisier datasets like Colon and Leukemia, Euclidean matrices appear disordered, failing to reveal consistent feature relationships. One minus correlation distance yields mixed outcomes; while it reveals some block structures in datasets like Ovarian and Prostate, it generally captures linear dependencies rather than continuous spatial gradients, deviating from the true model. In contrast, Geodesic distance produces smoother transitions and clearer diagonal formations in Ovarian and Prostate, indicating its ability to uncover intrinsic geometric structures. Jensen-Shannon distance demonstrates variability: it shows some spatial coherence in Prostate but generates fragmented and noisy patterns in Colon, Leukemia, and Ovarian, likely due to unstable distribution estimates in sparse data. Wasserstein distance shows strong performance in datasets like Colon, Leukemia, and Prostate, maintaining smooth, block-like rank structures. However, in ARCENE, the matrices become less coherent, likely due to sample noise and the difficulty of estimating reliable empirical distributions. The Tropical distance generates rank matrices with distinct structural characteristics; for the Prostate dataset, it produces a highly coherent diagonal pattern similar to the Euclidean and Geodesic metrics, suggesting it successfully captures the hierarchical dominance relationships in this data. However, for the Colon dataset, the Tropical distance matrix appears more fragmented, indicating that the max-plus metric structure may be less suitable for this specific gene expression profile.

Overall, Wasserstein distance most closely approximates the spatial layout of the true model across well-behaved datasets, offering more faithful and interpretable representations of feature relationships. While Euclidean and correlation-based metrics are more limited in capturing nonlinear structure. The performance between Geodesic distance and Jensen-Shannon distance are similar. Tropical distance shows promise in datasets with specific hierarchical structures but exhibits variability in others. These findings emphasize the value of integrating non-Euclidean distances, particularly Geodesic, Wasserstein, and Tropical into the IGTD framework, enhancing its ability to transform tabular data into meaningful spatial representations for CNN-based learning, while also revealing its boundaries in challenging scenarios.

### Pseudo-image visualization

**Fig 3:**
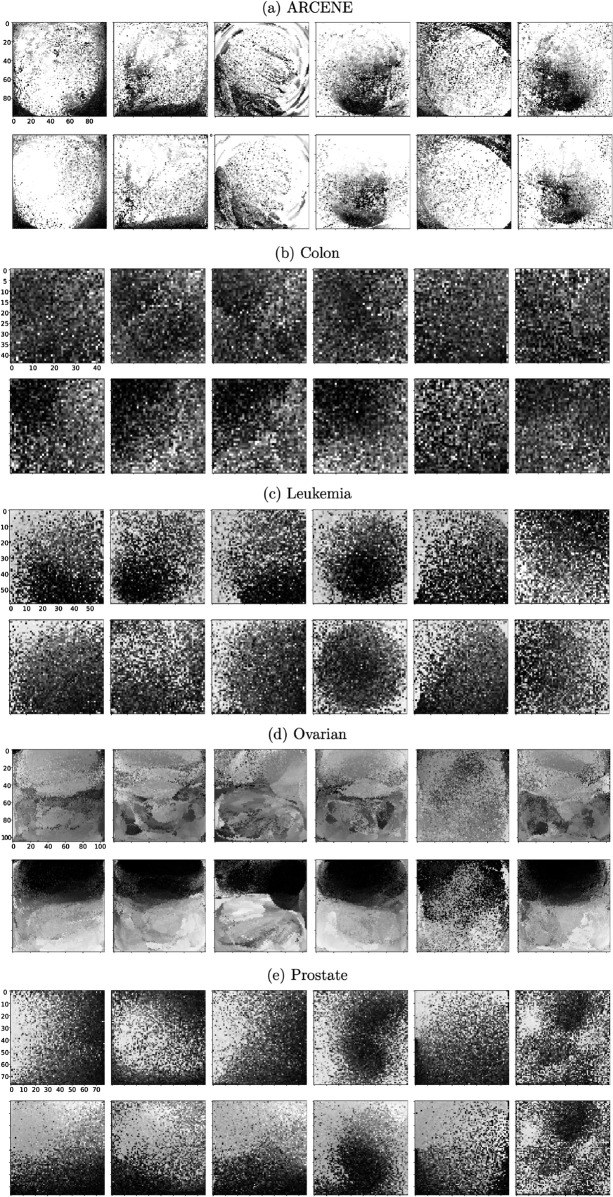
Representative pseudo-images for two classes under varied distance metrics. The first row displays pseudo-images from class 0, while the second row shows pseudo-images from class 1, for representative samples from each of the five datasets. Images are generated using six distance metrics, Euclidean distance, one minus correlation, Geodesic distance, Jensen-Shannon distance, Wasserstein distance, and Tropical distance, arranged from left to right.

Fig 3 examines the quality and structure of pseudo-images generated from tabular data for binary classification tasks using IGTD framework. For each of the five datasets, representative samples from class 0 and class 1 are visualized as grayscale images. These images were generated using six different distance metrics, and arranged from left to right as follows: Euclidean distance, one minus correlation distance, Geodesic distance, Jensen-Shannon distance, Wasserstein distance, and Tropical distance.

In ARCENE, pseudo-images generated using one minus correlation, Geodesic, Jensen-Shannon, Wasserstein distances, and Tropical distance revealed special patterns with clear class separability, in contrast to the diffuse, less structured patterns under Euclidean. This highlights the superiority of non-Euclidean metrics in capturing underlying feature geometry. In the Colon dataset, where the sample size is small and dimensionality high, all images appeared noisy, but Geodesic and Jensen-Shannon distances still produced marginally class separability, suggesting a slight advantage even under data constraints. The Leukemia dataset further emphasized the strength of Geodesic, Wasserstein, and Tropical metrics, which yielded more centered and structured intensity distributions, revealing clearer distinctions between classes. Euclidean distance again failed to preserve meaningful structure. In the Ovarian dataset, which benefits from a larger sample size and variable numbers, all distance metrics produced the most visually distinct class patterns. Finally, for Prostate, class differences were more distinct for Euclidean, one minus correlation, Geodesic and Jensen-Shannon distance metrics.

These visual comparisons demonstrate that non-Euclidean metrics such as Geodesic, Wasserstein, and Tropical distances consistently outperformed others in generating coherent and class-discriminative pseudo-images, regardless of sample size or feature complexity. These visual findings align with quantitative classification results, reinforcing the utility of non-Euclidean metrics in enhancing CNN-based learning from high-dimensional tabular data.

### Classification performance evaluation

Ten-fold cross-validation classification results (mean validation indices with one standard deviation) across five real-world datasets using six different distance metrics are given in Table 3. Overall, the results demonstrate that non-Euclidean metrics often outperform the baseline Euclidean distance in terms of accuracy and predictive performance. In the ARCENE dataset, IGTD(GD) achieved the highest accuracy (0.8650), outperforming IGTD(ED) (0.8150), with IGTD(WD) also showing strong performance (0.8500). The Colon dataset exhibited a notable increase in accuracy with IGTD(GD) (0.8357), compared to only 0.7452 for IGTD(ED). For Leukemia, IGTD(RD) and IGTD(JD) yielded higher accuracy scores (0.9464 and 0.9429, respectively) than the Euclidean-based method. In the Ovarian dataset, all methods performed exceptionally well, but IGTD(GD) and IGTD(RD) slightly surpassed IGTD(ED), with accuracy values approaching 0.99. For the Prostate dataset, IGTD(TD) utilizing Tropical distance achieved the highest accuracy (0.8718) and precision (0.9133) among all metrics, surpassing IGTD(RD) (0.8609) and IGTD(ED) (0.8227). This significant result underscores the effectiveness of tropical geometry in capturing the specific feature relationships inherent in prostate cancer genomic data. Conversely, IGTD(TD) showed variable performance across other datasets, such as Colon (0.5833), suggesting its utility may be domain-specific.

**Table 3:** Ten-fold cross-validation classification results (mean with one standard deviation) across five real-world datasets using six different distance metrics within the IGTD framework.

(a) ARCENE						
VIs	IGTD(ED)	IGTD(RD)	IGTD(GD)	IGTD(JD)	IGTD(WD)	IGTD(TD)
Accuracy	0.8150 (0.1001)	0.8100 (0.0539)	0.8650 (0.0610)	0.8050 (0.0650)	0.8500 (0.0632)	0.8250 (0.0559)
Precision	0.8161 (0.1056)	0.7934 (0.0767)	0.8381 (0.0754)	0.8148 (0.0970)	0.8465 (0.0768)	0.7794 (0.0521)
Recall	0.7417 (0.1971)	0.7861 (0.1023)	0.8403 (0.0759)	0.7264 (0.1258)	0.8083 (0.1099)	0.8431 (0.0883)
F1-Score	0.7651 (0.1654)	0.7833 (0.0010)	0.8367 (0.0612)	0.7616 (0.0865)	0.8232 (0.0772)	0.8085 (0.0619)
**(b) Colon**						
VIs	IGTD(ED)	IGTD(RD)	IGTD(GD)	IGTD(JD)	IGTD(WD)	IGTD(TD)
Accuracy	0.7452 (0.1502)	0.6952 (0.2176)	0.8357 (0.1293)	0.7429 (0.1498)	0.7357 (0.1870)	0.5833 (0.1737)
Precision	0.9000 (0.1658)	0.7967 (0.3143)	0.9250 (0.1460)	0.9350 (0.1343)	0.8083 (0.1665)	0.6588 (0.3679)
Recall	0.6750 (0.1953)	0.6500 (0.3000)	0.8250 (0.1146)	0.6750 (0.1953)	0.8250 (0.1601)	0.5250 (0.3437)
F1-Score	0.7595 (0.1718)	0.6889 (0.2881)	0.8679 (0.0972)	0.7607 (0.1330)	0.8058 (0.1358)	0.5449 (0.3070)
**(c) Leukemia**						
VIs	IGTD(ED)	IGTD(RD)	IGTD(GD)	IGTD(JD)	IGTD(WD)	IGTD(TD)
Accuracy	0.9018 (0.1281)	0.9464 (0.0864)	0.9196 (0.1404)	0.9429 (0.1309)	0.9286 (0.0958)	0.8875 (0.1782)
Precision	0.8667 (0.2082)	0.9667 (0.1000)	0.9000 (0.3000)	0.9400 (0.1800)	0.8333 (0.3073)	0.8833 (0.2363)
Recall	0.9333 (0.1333)	0.8833 (0.2363)	0.8000 (0.3999)	0.9667 (0.1000)	0.8667 (0.3055)	0.9333 (0.1333)
F1-Score	0.8800 (0.1514)	0.8967 (0.1716)	0.8300 (0.3164)	0.9371 (0.1357)	0.8400 (0.2939)	0.8767 (0.1700)
**(d) Ovarian**						
VIs	IGTD(ED)	IGTD(RD)	IGTD(GD)	IGTD(JD)	IGTD(WD)	IGTD(TD)
Accuracy	0.9762 (0.0319)	0.9920 (0.0160)	0.9922 (0.0157)	0.9802 (0.0267)	0.9760 (0.0265)	0.9802 (0.0199)
Precision	0.9882 (0.0237)	1.0000 (0.0000)	1.0000 (0.0000)	0.9941 (0.0176)	0.9879 (0.0243)	0.9824 (0.0270)
Recall	0.9750 (0.0415)	0.9875 (0.0250)	0.9879 (0.0243)	0.9754 (0.0411)	0.9750 (0.0306)	0.9879 (0.0243)
F1-Score	0.9810 (0.0257)	0.9935 (0.0129)	0.9937 (0.0125)	0.9840 (0.0219)	0.9810 (0.0209)	0.9847 (0.0154)
**(e) Prostate**						
VIs	IGTD(ED)	IGTD(RD)	IGTD(GD)	IGTD(JD)	IGTD(WD)	IGTD(TD)
Accuracy	0.8227 (0.1082)	0.8609 (0.1024)	0.8236 (0.1384)	0.8418 (0.1195)	0.8118 (0.0961)	0.8718 (0.1011)
Precision	0.8524 (0.1332)	0.9050 (0.1229)	0.8483 (0.1596)	0.8717 (0.1370)	0.8424 (0.1132)	0.9133 (0.1085)
Recall	0.8033 (0.1269)	0.8233 (0.1399)	0.8233 (0.1660)	0.8267 (0.1583)	0.7833 (0.1408)	0.8267 (0.1397)
F1-Score	0.8211 (0.1103)	0.8540 (0.1077)	0.8253 (0.1382)	0.8380 (0.1217)	0.8043 (0.1050)	0.8638 (0.1124)

In terms of stability, measured through standard deviations, IGTD(GD) consistently maintained a good balance between high accuracy and moderate variability, indicating robustness in modeling nonlinear structures. While IGTD(RD) and IGTD(JD) sometimes exhibited higher variance, particularly in smaller or more imbalanced datasets like Colon and Leukemia, IGTD(WD) generally combined strong performance with lower variability. These observations suggest that Wasserstein and Geodesic distances are both effective and reliable for capturing intrinsic relationships in high-dimensional feature spaces.

The influence of dataset characteristics, such as sample size and number of features, is also apparent in the results. Smaller datasets with high dimensionality, such as Colon (*n* = 62, *p* = 2000) and Leukemia (*n* = 72, *p* = 3571), showed the most pronounced performance gains when non-Euclidean metrics were used, particularly IGTD(GD). These improvements likely stem from the ability of non-Euclidean metrics to capture complex, nonlinear dependencies that traditional Euclidean distance may fail to detect.

These empirical findings underscore the critical importance of spatial coherence in the transformed data. The relevance of aligning with the theoretical true model extends beyond visual interpretability; it is fundamental to the mechanics of CNN-based classification. CNNs rely on the principle of locality, utilizing convolutional kernels to extract features from spatially adjacent pixels within a receptive field. A feature distance rank matrix that approximates the smooth, continuous structure of the true model indicates that the chosen distance metric has successfully mapped intrinsically similar features to neighboring coordinates on the image grid. This spatial coherence ensures that the CNN’s filters can effectively capture local correlations and structural patterns. Conversely, a fragmented or disordered rank matrix implies that related features remain scattered across the grid, negating the advantages of the convolutional architecture and hindering the model’s ability to extract robust representations.

## Conclusion and discussion

This study presents significant advancements to the Image Generator for Tabular Data (IGTD) framework by introducing non-Euclidean distance metrics, specifically one minus correlation distance, Geodesic distance, Jensen-Shannon distance, Wasserstein distance, and Tropical distance, to enhance the classification of high-dimensional tabular data using Convolutional Neural Networks (CNNs). Through the simulation studies and the extensive evaluation across five real-world benchmark datasets, we demonstrate that replacing the conventional Euclidean distance with more expressive non-Euclidean alternatives substantially improves classification accuracy, image quality, and the ability to capture complex feature structures.

Our findings, supported by classification performance, distance rank matrix analysis, and pseudo-image visualization, show that Geodesic and Wasserstein distances consistently outperform other metrics. Furthermore, Tropical distance demonstrated superior performance on the Prostate dataset, offering a unique advantage for specific genomic data structures. These methods produce spatially coherent image representations that preserve intrinsic geometric or distributional relationships among features, making them particularly well-suited for CNN-based learning. The improved fidelity of these image representations allows CNNs to extract more meaningful patterns, thereby enhancing overall model performance.

Beyond predictive gains, the refined IGTD framework offers methodological contributions by serving as a bridge between traditional statistical modeling and deep learning. It enables high-dimensional tabular data to be encoded into structured visual formats, which not only benefit CNN training but may also act as a form of informed data augmentation, capturing nonlinearity and spatial dependencies often missed by Euclidean-based transformations.

However, this study also highlights important limitations. Notably, the computational cost of metrics like Jensen-Shannon and Wasserstein distances can be substantial, particularly for large-scale or real-time applications, due to the complexity of estimating probability distributions and solving optimal transport problems. Although these metrics are computationally more demanding than the Euclidean distance, their added cost constitutes only a small portion of the total processing time, which is largely dominated by the subsequent CNN training and classification stages. Additionally, in highly sparse or noisy datasets, none of the distance metrics, including the best-performing ones, produced fully coherent representations, suggesting potential limitations of the IGTD framework in extreme data settings. The challenge of processing noisy or sparse tabular data for image conversion remains a fundamental limitation in most existing tabular-to-image deep learning methods. Currently, little research has explored how to effectively transform such imperfect tabular data into meaningful image representations without compromising critical structural patterns. Addressing this challenge presents a key opportunity for future work, as robust conversion techniques for noisy and sparse datasets could greatly enhance the real-world applicability of image-based tabular data classification.

To contextualize our findings, we compared our results against available state-of-the-art (SOTA) benchmarks identified through a survey of recent literature. For the Ovarian cancer dataset, our best-performing model achieved an accuracy of 99.2%, which is highly comparable to the 98.6% accuracy reported by [59] using a hybrid ReliefF-CNN model. However, for other datasets, highly specialized methods employing advanced feature selection often achieve higher absolute performance. For instance, while our method achieved approximately 94.6% on the Leukemia dataset, recent work by [60] reported 100% classification accuracy using optimized Support Vector Machine (SVM) and Logistic Regression models. Similarly, Asad and Mollah (2021) [61] reported 100% accuracy on the Colon dataset using Symmetrical Uncertainty-based feature selection with Random Forest and Multilayer Perceptron (MLP) classifiers, surpassing the 83.6% achieved by our IGTD(GD) model without feature selection. Comparable 100% accuracy benchmarks have also been reported for the Prostate dataset by [62], and accuracies reaching 99% have been achieved for ARCENE using the Enhanced Incremental Deep Multi-Layer Perceptron (EIDMLP) classifier [63]. It should be noted that while multiple studies in the literature may report similar high-performance metrics (e.g., 100% accuracy), we have prioritized citing the most recent and representative examples.

It is crucial to note that these external results serve primarily as reference points rather than direct benchmarks. Variations in data preprocessing (e.g., normalization techniques, outlier removal), feature selection methods (which alter the input dimensionality), and validation protocols (e.g., different cross-validation folds or train/test splits) mean that the same dataset name often refers to slightly different experimental inputs in the literature. Furthermore, this performance disparity is a natural consequence of our experimental scope; our primary objective was to isolate and evaluate the specific impact of distance metrics on tabular-to-image conversion, rather than to engineer a maximally optimal classification pipeline. To ensure a rigorous internal comparison, we deliberately employed a standardized, fixed CNN architecture across all experiments without extensive hyperparameter tuning or advanced feature selection techniques, factors that are often critical drivers of SOTA performance. Consequently, while our absolute accuracies are lower than highly specialized models, the relative performance gains observed with non-Euclidean metrics confirm their utility.

Future research may focus on developing more scalable implementations of complex distance metrics, such as approximations of optimal transport or kernel-based geodesic computations, to reduce computational overhead. Additionally, exploring adaptive metric selection strategies, where the most suitable distance function is learned from the data rather than predetermined, could offer valuable improvements. Furthermore, integrating ED, RD, GD, JD, WD, and TD as stages of data augmentation within the CNN pipeline may lead to enhanced performance and greater interpretability.

In conclusion, the integration of non-Euclidean metrics within the IGTD framework significantly enhances its ability to transform tabular data into meaningful image representations for deep learning. These findings underscore the importance of selecting appropriate distance functions for tabular-to-image conversion and affirm the potential of non-Euclidean IGTD as a robust, interpretable, and versatile tool for CNN-based classification of complex tabular datasets.

## Supporting information

S1 Code R and Python source code. (ZIP)
